# Yeast-powered microfluidic pump based on a four-parameter fermentation model

**DOI:** 10.1038/s41378-026-01294-1

**Published:** 2026-05-15

**Authors:** Jeongmok Kim, Kideok Kim, Seongyeol Baeck, Joong Yull Park

**Affiliations:** 1 School of Mechanical Engineering, College of Engineering, Chung-Ang University, Seoul, Republic of Korea; 2 Department of Intelligent Energy and Industry, Graduate School, Chung-Ang University, Seoul, Republic of Korea

**Keywords:** Engineering, Chemistry

## Abstract

Baker’s yeast is a common microorganism that is well-known for its fermentation activities. The fermentation process naturally produces CO_2_, leading to a gradual increase in internal pressure within sealed environments. Meanwhile, passive pumps are rising in the microfluidics field for their simplicity, low energy requirements, and suitability for portable and disposable devices. Here, we harnessed yeast fermentation as a biological power source for a passive pump, enabling fluid flow in microfluidic systems. This approach introduces a cost-effective solution and extends the concept of passive pumping into the realm of biological systems. The custom mechanical pump operates by converting the gas pressure generated by fermentation into continuous fluid movement. The dynamics of gas production within the pump were analyzed experimentally to characterize performance over time. The resulting six-parameter and four-parameter equations accurately capture the experimental trends within the validated operating range. This model was further extended to a simplified two-parameter form—using only yeast mass and sucrose concentration—making the pump setup more intuitive. This biologically driven pump concept holds potential for expansion into autonomous microfluidic devices, especially for space orbital experiment modules, educational tools, or low-resource settings where external power sources are limited.

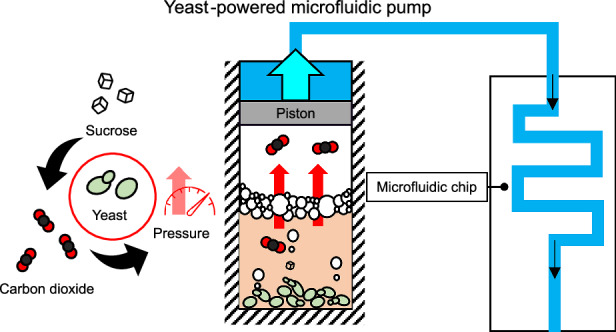

## Introduction

### Concept of passive pumping

Microfluidics holds great potential for the analysis and experimentation of biological and chemical systems. From wearable and handheld devices to compact experimental platforms, microscale fluid flow has demonstrated significant utility across a wide range of applications. These systems typically require flow rates of only a few microliters per minute. Achieving such low flow rates necessitates the use of micropumps, such as rotary, syringe, or diaphragm pumps, which rely on electricity. However, electrically powered micropumps present several drawbacks, including their dependence on external power sources, bulky design, and high cost. To address these limitations, extensive research has focused on developing alternative pumping mechanisms that do not require external power or that can be directly integrated into microfluidic chips to enhance portability and design simplicity. These alternatives are referred to as passive pumps. Examples of passive pumping technologies include surface tension^1^, electrolysis^2^, vacuum-driven flow^3–5^, gravity-driven flow^6,7^, osmotic pressure^8–11^, capillary force^12,13^, evaporation-driven flow^13,14^, thermal expansion^15^, air-infused PDMS^16^, and gas-producing chemical reactions such as acid-base neutralization (CO_2_ generation)^17,18^ or hydrogen peroxide decomposition (O_2_ generation)^19^. These passive pumps have proven effective in various applications and offer practical advantages for low-cost, compact microfluidic systems. Beyond these physical and chemical approaches, biological activity has also been employed to generate fluid flow. For example, enzymatic reactions can create local density gradients by catalyzing substrate conversion, which induces convective flow^20–22^. However, enzyme-based systems typically produce only localized flow and generate weaker forces compared to electrically powered micropumps, limiting their practical applications.

### Harnessing yeast into passive pumps

Yeast, specifically *Saccharomyces cerevisiae*, is a well-known microorganism that is also suitable for driving passive microfluidic pumps. It possesses several characteristics that make it an excellent candidate for biological actuation. First, yeast is highly pressure-resilient; it not only tolerates elevated pressures but also generates pressure during fermentation. This enables the yeast-driven pump to deliver sufficient force to overcome flow resistance in complex or branched microfluidic channels, where substantial pressure may be necessary. Second, the materials required to operate the system are extremely low-cost. Assuming the pump is reusable following sterilization, the material cost per experiment is estimated at less than $0.01 USD per 1 mL of yeast medium. This affordability is made possible by the use of commercially available instant yeast and sucrose. Furthermore, yeast can function under anaerobic conditions, allowing the pumping system to be isolated from the buffer fluid that flows through the microsystem. Such isolation is effective both mechanically and biologically. For efficient pumping, gas generated inside the pump must be sealed to prevent leakage. From a biological side, yeast spreading outside the pump could be a concern. As a result, we selected a syringe to separate the yeast chamber from the pump reservoir.

**Fig. 1:**
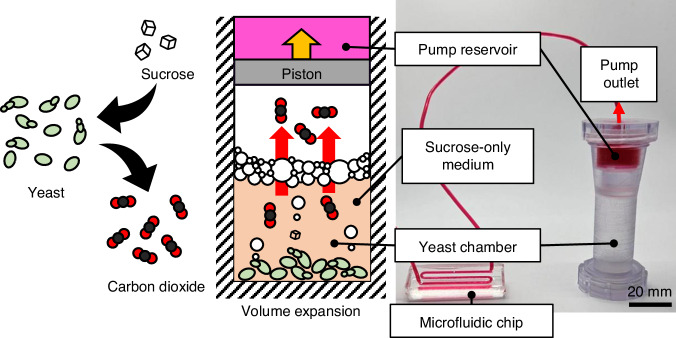
Concept diagram and photo of the yeast-powered pump. The pump design traps both yeast and gas within a sealed chamber, resulting in a gradual buildup of internal pressure. This pressure drives a piston, displacing the liquid medium from the reservoir through the outlet and into a connected microfluidic platform. The system relies solely on water, sucrose, and instant yeast—materials that are simple, inexpensive, and widely available

Inspired by the characteristics of *Saccharomyces cerevisiae*, we introduce a novel bio-driven pump that harnesses the fermentation activity of yeast to generate pressure—termed the yeast-powered pump, or simply, the yeast pump. This device can sustain continuous fluid flow for several hours to days without external power and is compact enough to be held in one hand, making it highly suitable for portable or incubator-based applications (Fig. 1).

## Results and discussion

### Selecting pump configuration options

*Saccharomyces cerevisiae* is a well-studied microorganism with extensive literature describing the dynamics and environmental dependencies of its fermentation. Yeast fermentation is highly sensitive to factors such as temperature, pH, carbon source, inoculum size, free amino nitrogen (FAN), vitamins, and minerals^23^. These sensitivities mean the yeast pump can be configured in many ways, but they also motivate simplifying choices when the device is intended as a practical, customized tool. Among the controllable variables, inoculum mass, substrate concentration, and temperature are most commonly used to manipulate fermentation dynamics. Temperature is particularly influential and difficult to manage passively. For example, lowering the temperature from 36 °C to 25 °C can reduce metabolic rate by roughly twofold (Supplementary Fig. S1). Temperature was therefore treated as a constant, and the pump assemblies were thermally insulated. For substrate, a simple sucrose solution was chosen as the primary medium, providing a controlled and consistent substrate for fermentation. Moreover, this streamlined medium setup reduces both the complexity and preparation time, allowing the yeast pump to function more as a practical tool than a biological culture. To further enhance this convenience, instant dry yeast was selected for its ease of use, long shelf life, and rapid activation. Combined with a sucrose solution, which also offers extended storage stability, the system is highly accessible and ready for immediate deployment.

**Fig. 2:**
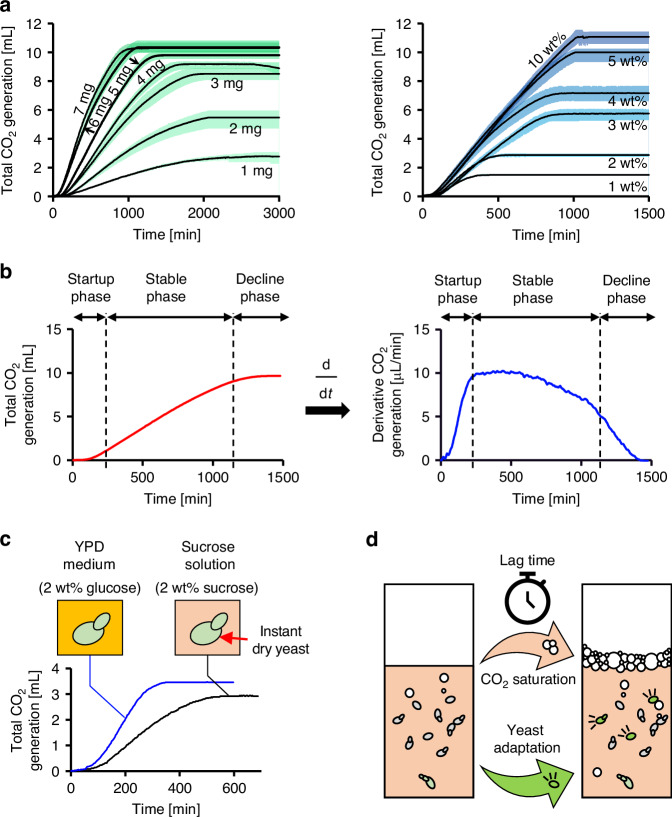
Phases of yeast formation and CO_2_ production behavior for two major yeast pump configurations (yeast inoculum mass and sucrose concentration). **a** The performance of the yeast pump can be modulated by adjusting the ratio of yeast inoculum to sucrose concentration. Shaded regions represent the range between the minimum and maximum values across six replicates (*N* = 6). **b** The plots illustrate three distinct phases of pump operation: startup, stable, and decline. Supplementary Movie S1 demonstrates the pump in operation. **c** A comparison between YPD and a simplified sucrose solution reveals similar trends in CO_2_ generation, supporting the use of the simplified medium for practical experimentation. **d** Illustration of the startup phase, representing the transition from initial inoculation to stable CO_2_ production, influenced by metabolic adaptation to the surrounding environment and CO_2_ accumulation in the medium to active gas release (effervescence)

To evaluate the relationship between gas production and this streamlined configuration, a preliminary case study was conducted (Fig. 2a, Table S1), analyzing the rate of gas generation during fermentation (Fig. 2b). The overall trend of CO_2_ generation closely resembles that observed in conventional fermentation studies, where yeast proliferates exponentially in yeast extract peptone dextrose (YPD) medium (Fig. 2c). This similarity likely results from residual nutrients retained during the production of instant yeast, such as vitamins and trace minerals, which can sustain metabolic activity for one to two days. In this study, yeast was observed to sustain fermentation for at least one day using only a sucrose solution. Furthermore, it was confirmed that instant yeast cannot initiate fermentation in the absence of supplemental sucrose, validating the choice of sucrose as a key experimental parameter.

The standard condition for operating the yeast pump consists of 5 mg of instant yeast and 5 wt% sucrose dissolved in 1 mL of water. For example, the 3 mg inoculum case refers to 3 mg of yeast with 5 wt% sucrose in 1 mL of water, while the 2 wt% sucrose case involves 5 mg of yeast with 2 wt% sucrose in the same volume. Increasing the yeast inoculum mass resulted in a higher pumping rate but a shorter overall runtime. Compared to the standard case, the highest tested inoculum (7 mg) increased the pumping rate by up to 49.2% but reduced the total runtime by 33.8%. Conversely, the lowest inoculum (1 mg) caused an 83.5% decrease in pumping rate and extended the runtime by 57.3%. Adjusting the inoculum mass within the narrow range of 1–7 mg significantly influenced the performance trends of the pump, primarily because yeast proliferation is suppressed under the simplified nutrient conditions.

Reducing the sucrose concentration shortened the runtime of the pump and caused only a modest decrease in the pumping rate. Unlike the inoculum mass case, there was no clear trade-off between pumping rate and runtime. Compared to the standard condition, the 1 wt% sucrose case resulted in a 74.97% reduction in runtime, while the pumping rate decreased by only 13.03%. By contrast, the runtime for the 10 wt% sucrose cases could not be fully measured because it exceeded the CO_2_ volume detection limit, although the pumping rate increased by 31.82%.

In essence, the pumping rate is primarily determined by the yeast inoculum mass, while the total runtime can be adjusted by varying the sucrose concentration. Although both parameters influence runtime, an effective control strategy involves first establishing the target flow rate through inoculum mass, followed by fine-tuning the runtime by optimizing the sucrose level. Notably, excessively high sucrose concentrations can inhibit fermentation owing to osmotic stress, which typically occurs at approximately 30 wt% sucrose^24^.

### Yeast pump model development

#### Analysis of pump phases

The fermentation rate of yeast is the key parameter for analysis. Although conventional models such as Monod and Gompertz equations provide useful insights, they are limited in capturing the full fermentation process from initiation to cessation in this pump. For instance, logistics, Monod, and Morgan-Mercer-Flodin models fail to capture the initial lag phase observed at the beginning of fermentation. During the middle phase, CO_2_ production in the yeast pump exhibits a more linear trend than predicted by these traditional models. Moreover, the ending phase in this system does not exhibit a gradual tapering off; instead, it features a sudden drop in gas production, with the rate of CO_2_ generation decreasing abruptly before coming to a complete stop. More detailed comparison between the yeast pump and the linear pump is shown in Supplementary Fig. S2. These distinct behaviors highlight the need for a customized modeling approach. To achieve more accurate predictions, a customized mathematical model was deployed, combining features of existing models. This required a detailed investigation of yeast growth kinetics in a sucrose solution, leading to the formulation of a new fitting model that effectively represents the dynamic behavior of the yeast pump. This model incorporates key features observed in our system, including the startup delay, diauxic shift, and extended steady-phase operation, and is subsequently simplified for practical use.

The yeast pump operates in three distinct phases: startup, stable, and decline phases (Fig. 2b). Each phase arises from underlying biological and physical processes. The startup phase represents the period during which the yeast system prepares for consistent CO_2_ generation. During this initial stage, the dried yeast must first rehydrate and activate, followed by a biological adaptation to the surrounding medium. This delay, commonly referred to as the lag phase, has been widely studied, and various models have been developed to explain its causes and influencing factors. In parallel, the liquid medium must reach CO_2_ saturation to support stable gas release, a process governed by both diffusion and convective transport. In addition, it is ideal to control dissolved O_2_ in the media, as yeast should ferment under anaerobic conditions. Although dissolved O_2_ could cause the startup phase, it was not actively controlled in this study, as deoxygenation is challenging, and the small to medium volume (1 mL) would rapidly equilibrate with atmospheric oxygen during mixing and handling of yeast. Together, these biological and physical factors determine the time required for the system to stabilize and reach steady-state operation (Fig. 2d). Because this phase is derived from the lagging phase, removing or shortening this phase would be advisable. However, pre‑activation by prebatching or by pressurizing the chamber did not reliably shorten startup in our tests, and resolving this will require more detailed investigation. Consequently, we treat the startup delay as an intrinsic aspect of the pump’s performance in the present study.

The stable phase is marked by continuous CO_2_ production, during which the yeast ferments sugars at a relatively consistent rate. The pumping rate typically peaks at the beginning of this phase, reflecting the maximum metabolic activity of the yeast. After this peak, the rate gradually declines as the substrate concentration decreases and inhibitory byproducts—such as ethanol and carbonic acid—accumulate. Despite this moderate decline in output, the system maintains a relatively steady flow, making this phase the most efficient period for fluid delivery. This stage reflects a temporary metabolic equilibrium under constrained environmental conditions.

The decline phase marks the gradual reduction in pump activity. Post-experiment viability tests confirm that yeast cells retain over 99% viability during this phase (Fig. S3**, Supplementary Information**), indicating that the observed decline is not due to cell death. Instead, it reflects a metabolic downshift driven by deteriorating environmental conditions—specifically, elevated ethanol concentrations, reduced pH, and the depletion of readily fermentable sugars such as glucose and fructose. A significant drop in the pumping rate is observed at the transition from the stable to the decline phase. This abrupt change is attributed to a metabolic shift from glucose to fructose, which arises from the hydrolysis of sucrose. Because yeast metabolizes fructose at a slower rate than glucose, the decline phase likely begins when glucose becomes depleted.

#### Startup phase modeling

The startup phase of the yeast pump is influenced by a range of interrelated biological and physical factors, including the rehydration of dried instant yeast, the lag phase associated with metabolic adaptation, short-term exponential growth, and the progressive accumulation of CO_2_ within the chamber. Accurately isolating and modeling each of these components is highly complex and often functionally impractical owing to their overlapping effects and nonlinear interactions. Therefore, a simplified sigmoid function was employed to approximate the overall startup behavior.

Although not all individual factors are explicitly represented, the underlying physical principles—such as diffusion-driven CO_2_ absorption in water (typically modeled by an exponential curve)^25^, the hydration kinetics of dried cells (logistic curve)^26^, and early-phase yeast growth dynamics (sigmoid curve)^27^,—share common mathematical characteristics. Taking these shared dynamic trends into account, the startup phase is modeled using the following sigmoid function Eq. (1):

1 $${Q}_{{\rm{start}}}\left(t\right)={s}_{f}\frac{\exp {s}_{t}\left(t-{t}_{i}\right)}{1+\exp {s}_{t}\left(t-{t}_{i}\right)}$$

where *Q*_*start*_(*t*) denotes the estimated yeast-driven pumping activity over time. The parameter *s*_*f*_ is a scaling factor that determines the peak activity level, *s*_*t*_ controls the steepness of the sigmoid curve, and *t*_*i*_ represents the latency corresponding to the startup delay. This function governs the time-dependent pumping behavior during the startup phase.

#### Stable phase modeling

**Fig. 3:**
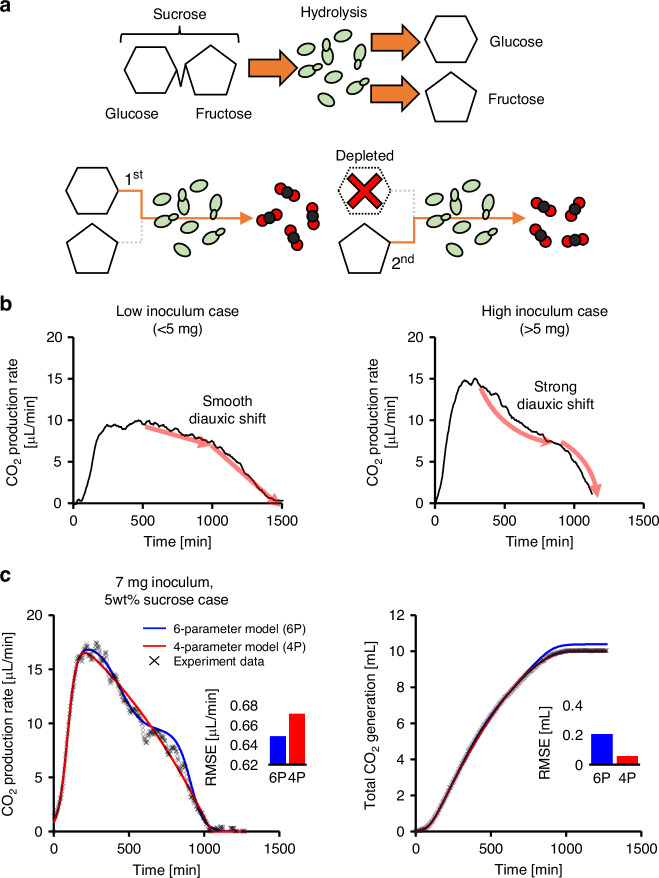
Concept of the diauxic transition and its effect on production rate fluctuations and two types of curve-fitting models. **a** Yeast enzymatically hydrolyzes sucrose into glucose and fructose, producing a medium with multiple carbon sources. Because glucose is metabolized more readily, yeast first consumes glucose and then transitions to fructose. **b** The diauxic shift temporarily reduces CO_2_ production and appears as a dip or fluctuation in the CO_2_ production rate curve. Higher yeast inoculum levels accentuate the diauxic shift, resulting in more pronounced fluctuations. **c** Two mathematical models were fitted to experimental data to evaluate accuracy. The blue curve incorporates the diauxic shift and closely aligns with the CO_2_ production rate trend. In contrast, the red curve omits the diauxic shift and provides a more accurate fit for cumulative CO_2_ volume. The data shown correspond to the 7 mg inoculum condition

In the context of the yeast pump, the transition between the stable and declining phases is gradual, with no distinct inflection point separating them. As fermentation progresses, the available carbon sources in the medium become depleted, leading to a decline in metabolic activity (Fig. 3a). Under these nutrient-limited conditions, yeast cells begin to enter a quiescent state^28,29^. During this phase, yeast preferentially consumes glucose, as it is more readily metabolized. Because sucrose is enzymatically hydrolyzed into glucose and fructose, a metabolic shift occurs once glucose is exhausted—a phenomenon commonly referred to as the diauxic shift. Representative data for the 3 mg and 6 mg inoculum cases are shown in Fig. 3b, while the complete datasets for all tested inoculum levels (1–7 mg) and sucrose concentrations (1–5 wt%) are provided in **the Supplementary Information** (Figs. S4, S5). This metabolic transition results in fluctuations in the pump rate, as the yeast adapts to metabolize fructose.

Although modeling the diauxic shift remains an underdeveloped area, some studies have approached it by combining two sigmoid functions using a weighting mechanism^30^. Building on this concept, the diauxic behavior in the yeast pump was modeled using a logistic function Eq. (2):

2 $${Q}_{{stable}}\left(t\right)=\left(1+\frac{1}{1+\exp \frac{t-{t}_{s}}{{t}_{{ds}}}}\right)$$

where *t*_*s*_ denotes the time at which the diauxic shift begins, and *t*_*ds*_ controls the steepness of the transition. A higher *t*_*s*_ indicates a delayed onset of the shift, while a larger *t*_*ds*_ reflects a more pronounced and abrupt change in the pump rate.

#### Decline phase modeling

The decline phase represents the final stage of yeast pump operation, during which the system’s metabolic activity gradually ceases due to complete nutrient depletion and an increasingly unfavorable environment. During this period, yeast cells stop producing CO_2_ and enter a quiescent state. Notably, cell viability remains above 99% even after pump activity ceases, indicating that this phase is not a true “death phase” but rather a state of metabolic dormancy.

The final carbon source—fructose—is consumed in an exponentially decaying manner, and the pump’s declining performance can be modeled using the following Eq. (3):

3 $${Q}_{{decline}}\left(t\right)=\max \left(0,e-\exp \left(\frac{t}{{t}_{e}}\right)\right)$$

where *t*_*e*_ denote the time at which pump function ceases entirely.

To maintain consistent scaling, each phase fitting function is normalized to approach a value of 1 outside its effective range, ensuring it does not influence the pump rate during other phases. Consequently, the overall pump rate over time, *Q*_*total*_(*t*), is modeled as the product of the three phase functions Eq. (4):

4 $${Q}_{{total}}\left(t\right)={Q}_{{startup}}\left(t\right)\cdot {Q}_{{stable}}\left(t\right)\cdot {Q}_{{decline}}\left(t\right)$$

The complete expression for CO_2_ production rate can thus be written as Eq. (5):

5 $$\begin{array}{l}{Q}_{{total}}\left(t\right)={s}_{f}\frac{\exp {s}_{t}\left(t-{t}_{i}\right)}{1+\exp {s}_{t}\left(t-{t}_{i}\right)}\\ \left(1+\frac{1}{1+\exp \frac{t-{t}_{s}}{{t}_{{ds}}}}\right)\cdot \max \left(0,e-\exp \left(\frac{t}{{t}_{e}}\right)\right)\end{array}$$

And the integral form of cumulative CO_2_ production, *V*_*total*_(*t*), can be described as:

6 $$\begin{array}{l}{V}_{{total}}\left(t\right)={\int }_{0}^{t}{Q}_{{total}}\left(\tau \right){\rm{d}}\tau \\ ={\int }_{0}^{t}{s}_{f}\frac{\exp {s}_{t}\,\left(\tau -{t}_{i}\right)}{1+\exp {s}_{t}\left(\tau -{t}_{i}\right)}\cdot \left(1+\frac{1}{1+\exp \frac{\tau -{t}_{s}}{{t}_{{ds}}}}\right)\\ \cdot \max \left(0,e-\exp \left(\frac{\tau }{{t}_{e}}\right)\right){\rm{d}}\tau \end{array}$$

Although the yeast pump medium consists solely of sucrose and water—lacking any nitrogen-rich components such as those found in YPD—and yeast reproduction is strongly inhibited, the system still exhibits exponential behavior across all phases. This observation suggests that modeling pump performance should consider not only yeast population but also the productivity of individual cells under nutrient-limited conditions.

#### Simplifying the model

Although the fitting curve derived in Eq. (6) effectively represents the experimental production rate data, it relies on six fitting parameters: *s*_*f*_, *s*_*t*_, *t*_*i*_, *t*_*s*_, *t*_*ds*_, and *t*_*e*_, and is therefore referred to as the six-parameter model. To reduce model complexity and improve general usability, we identified the diauxic fitting term—responsible for capturing fluctuations in pump rate during the glucose-to-fructose metabolic transition—as a potential source of overfitting. Additionally, the high number of parameters makes the fitting process unstable and often necessitates manual tuning, reducing the consistency and reliability of the results. Therefore, this term was excluded to yield a simplified model with four parameters: *s*_*f*_, *s*_*t*_, *t*_*i*_, and *t*_*e*_, referred to as the four-parameter model. The equation can be further simplified by restricting the time domain to the interval 0 ≤ *t* ≤ *t*_*e*_; function values beyond *t*_*e*_ are not meaningful. The simplified model is presented as follows Eq. (7):

7 $${Q}_{{total}}\left(t\right)={s}_{f}\frac{\exp {s}_{t}\left(t-{t}_{i}\right)}{1+\exp {s}_{t}\left(t-{t}_{i}\right)}\left(e-\exp \left(\frac{t}{{t}_{e}}\right)\right)$$

As expected, the four-parameter model does not capture the “wiggle” behavior observed in the stable phase due to the diauxic shift (Fig. 3c), whereas the six-parameter model captures these short-term fluctuations. Quantitatively, the six-parameter model improved instantaneous rate fidelity. High-order curve fitting is expected to capture detailed trends; however, the accumulated error increased more than in the four-parameter model. The normalized root mean squared error (NRMSE) in the fitted pump rate increased slightly, from approximately 3.856% to 4.070%. However, the simplified curve (four-parameter model) provided better accuracy in modeling the cumulative CO_2_ volume, *V*_*total*_(*t*) (Fig. 3d), reducing the total fitting error from 1.997% to 0.587%. This result demonstrates a trade-off between capturing short-term dynamic fluctuations and achieving robustness and simplicity in modeling overall system behavior. Even though the four-parameter model was selected for this study because cumulative pumped volume was the primary focus, the six-parameter model remains advantageous when high temporal resolution of metabolic rate is required in specific fields, and its use may open new directions for research.

### Model parameters analysis

**Fig. 4:**
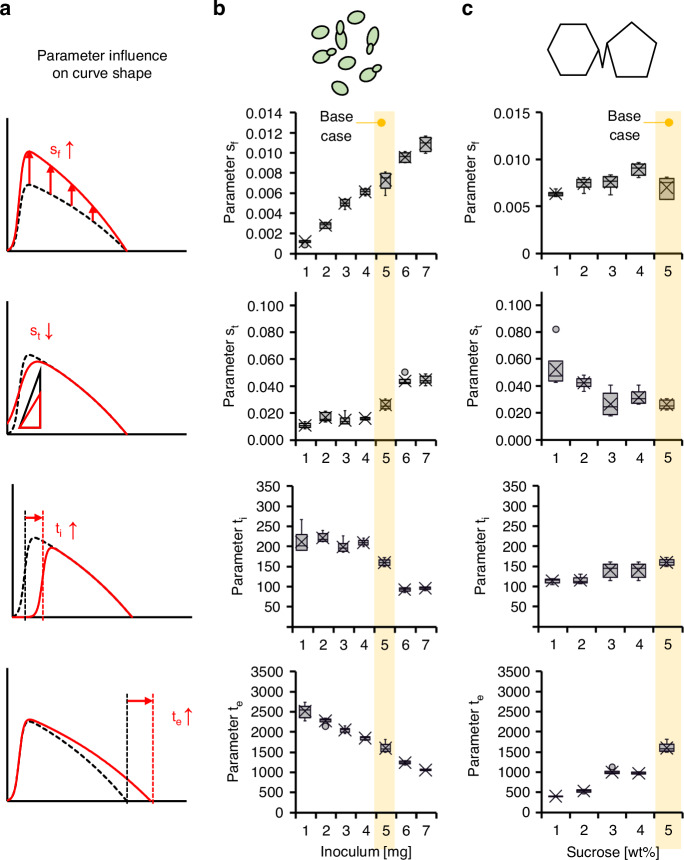
Parameter fitting results for yeast inoculum and sucrose concentration tests. **a** Parameter values for the four-parameters model—scaling factor (*s*_*f*_), steepness factor (*s*_*t*_), initiation time (*t*_*i*_), and exhaustion time (*t*_*e*_) —are shown for two experimental conditions: **b** varying yeast inoculum mass (1–7 mg, center), and **c** varying sucrose concentration (1–5 wt%, right). Each box plot displays the range of fitted parameter values for the respective yeast or sucrose condition, with error bars indicating variability across six replicates (*N* = 6). The yellow line denotes the base condition of 5 mg yeast and 5 wt% sucrose

Each experiment described in Fig. 2, which examined the effects of yeast mass and sucrose concentration on pump performance, was analyzed using the four-parameter model. The fitting process was performed in MATLAB using a custom-defined function and optimized using the linear least squares curve fitting method. The resulting parameters provide physical interpretations related to yeast activity and fermentation dynamics. Figure 4a illustrates how each parameter influences the shape and progression of the pump rate curve. Specifically, *s*_*f*_ governs the overall magnitude of CO_2_ production, *s*_*t*_ controls the steepness of the startup phase, *t*_*i*_ sets the latency or delay before activation, and *t*_*e*_ indicates the endpoint of pump operation. This analysis helps link the fitted mathematical model to the underlying biological behavior.

The dependence of each model parameter on yeast inoculum mass showed consistent trends (Fig. 4b). Increasing yeast mass produced a clear upward trend in the scaling factor *s*_*f*_, reflecting a higher maximum pump rate and confirming that CO_2_ output is primarily determined by active biomass. Similarly, *s*_*t*_, which controls the steepness of the startup curve, also increased, indicating a more rapid onset of CO_2_ production with greater yeast loads. By contrast, the latency parameter *t*_*i*_ decreased, suggesting that pumps with higher yeast content activate more quickly. The decline time parameter *t*_*e*_ also decreased, implying that higher yeast concentrations lead to earlier cessation of pump function, likely due to accelerated nutrient depletion. These parameter trends validate the biological underpinnings of the model and highlight how yeast mass influences both the intensity and duration of pump activity.

Each fitting parameter varies with changes in sucrose concentration, in contrast to the trends observed with the yeast inoculum mass case (Fig. 4c). The scaling factor *s*_*f*_ remains relatively stable across the tested sucrose range, indicating that the maximum pump rate is not strongly influenced by carbon supply when the yeast mass is fixed. However, the steepness parameter *s*_*t*_ and the latency parameter *t*_*i*_ show only slight variations, suggesting that startup kinetics are minimally affected. By contrast, the decline in the onset parameter *t*_*e*_ increases substantially with higher sucrose concentrations, reflecting prolonged pump operation due to extended carbon availability. Similarly, the parameter representing the duration of the decline phase also increases, indicating a more gradual and delayed shutdown. These trends confirm that sucrose concentration predominantly governs the runtime and termination behavior of the yeast pump without significantly affecting its peak flow rate.

Both test cases collectively demonstrate how yeast inoculum mass and sucrose concentration independently influence the fitted parameters of the simplified yeast pump model. Yeast mass exhibits strong correlations with all four parameters, *s*_*f*_, *s*_*t*_, *t*_*i*,_ and *t*_*e*_. By contrast, sucrose concentration primarily affects *t*_*e*_, without substantially altering the pump rate, as reflected in the relatively stable values of *s*_*f*_. However, it significantly influences the timing of the decline phase. These complementary trends support a control strategy in which yeast inoculum is selected to achieve the desired pump rate, followed by sucrose level adjustment to fine-tune the runtime, enabling independent modulation of performance characteristics through coordinated biological and chemical inputs.

### Two-parameter model interpolation and extrapolation

**Fig. 5:**
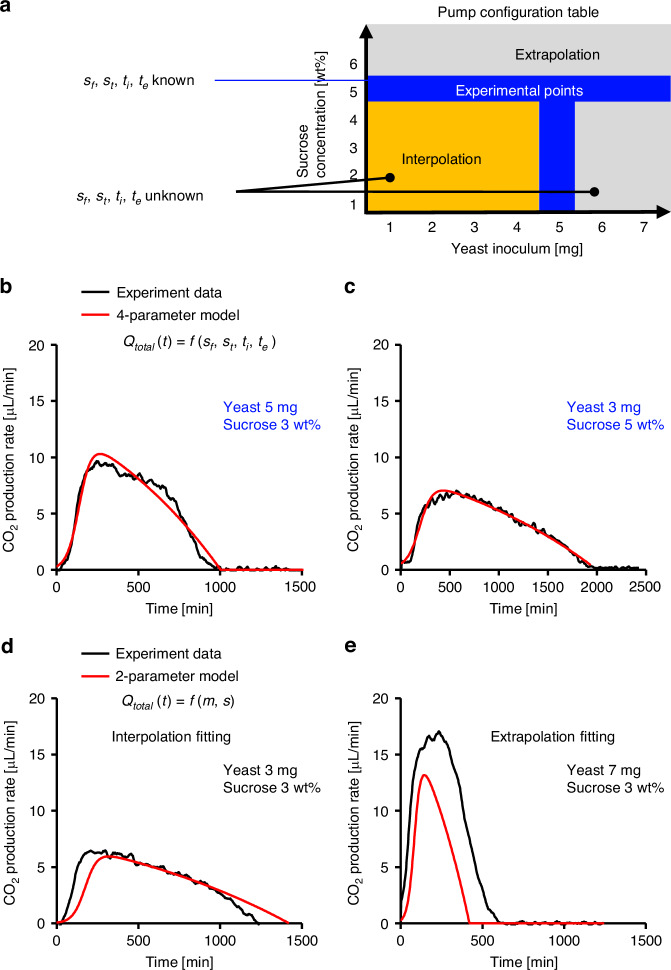
Comparison of four-parameter model fitting and two-parameter model interpolation and extrapolation. **a** Pump configuration chart showing the experimental setups. **b** The four-parameter model predictions versus averaged experimental results for 5 mg yeast with 3 wt% sucrose, and **c** for 3 mg yeast with 5 wt% sucrose, both within the original dataset, showing strong agreement. **d** The two-parameter model shows good agreement for interpolation cases, such as 3 mg yeast with 3 wt% sucrose. **e** However, it shows larger deviation under extrapolated conditions, such as 7 mg yeast with 3 wt% sucrose

Owing to the nonlinear nature of the pump rate function, determining the exact combination of yeast inoculum and sucrose concentration required to achieve a target pump rate and runtime is challenging, as all parameters depend on both variables. However, parameter values can be interpolated from Fig. 5a for chosen conditions, then used to calculate pump rate and runtime. Each of the four parameters can be expressed as a linear function of yeast mass (*m*) and sucrose concentration (*s*), a function even more reduced to the two-parameter model as shown in Eqs. (8–11) and Table 1:

8 $${s}_{f}\left(m,s\right)={C}_{m{s}_{f}}\cdot m+{C}_{s{s}_{f}}\cdot s+{C}_{{s}_{f}}$$

9 $${s}_{t}\left(m,s\right)={C}_{m{s}_{t}}\cdot m+{C}_{s{s}_{t}}\cdot s+{C}_{{s}_{t}}$$

10 $${t}_{i}\left(m,s\right)={C}_{m{t}_{i}}\cdot m+{C}_{s{t}_{i}}\cdot s+{C}_{{t}_{i}}$$

11 $${t}_{e}\left(m,s\right)={C}_{m{t}_{e}}\cdot m+{C}_{s{t}_{e}}\cdot s+{C}_{{t}_{e}}$$

**Table 1:** Coefficients for reducing a four-parameter model to a two-parameter linear fit

Coefficient	Value	Coefficient	Value	Coefficient	Value
*C*_*msf*_	0.0016218	*C*_*ssf*_	0.0002183	*C*_*sf*_	−0.0013759
*C*_*mst*_	0.0056424	*C*_*sst*_	-0.0043008	*C*_*st*_	0.022460
*C*_*mti*_	−22.551974	*C*_*sti*_	8.596592	*C*_*ti*_	217.890016
*C*_*mte*_	−250.45527	*C*_*ste*_	303.71105	*C*_*te*_	1262.644

The four-parameter model accurately predicts pump performance within the experimental range shown in Fig. 5b, c, where all parameters are known. For interpolated conditions—such as 3 mg yeast with 3 wt% sucrose, where four-parameters are unknown—the two-parameter model provides reliable predictions (Fig. 5d). Its main advantage is intuitiveness—the parameters are simply yeast mass and sucrose concentration—making pump configuration straightforward. However, extrapolation is unreliable. For example, at 7 mg yeast with 3 wt% sucrose, predicted and measured performance deviate noticeably (Fig. 5e). Therefore, the model should be applied only for yeast masses less than 5 mg and sucrose concentrations less than 5 wt%, with caution beyond these limits.

Rather than calculating the pump rate over the entire time range, the time of maximum performance and its corresponding pump rate can be determined by applying derivatives with suitable assumptions, as shown in Eqs. (12) and (13). The derivation and underlying assumptions are presented in Eq. S1:

12 $${t}_{\max }={t}_{i}+\frac{1}{{s}_{t}}{\mathrm{ln}}\left({s}_{t}{t}_{e}-1\right)$$

13 $${Q}_{\max }={Q}_{{total}}\left({t}_{\max }\right)$$

Here, *t*_*max*_ represents the time of maximum performance, and *Q*_*max*_ denotes the peak pump rate. For example, in the base case using 5 mg of yeast and 5 wt% sucrose, the fitted parameters *s*_*f*_, *s*_*t*_, *t*_*i*_ and *t*_*e*_ were 0.007291, 0.026052, 159.6 and 1599.6 respectively, as determined from the parameter chart (Fig. 4). For this configuration, the estimated runtime (*t*_*e*_) is 1599.6 min, the time to peak pump rate (*t*_*max*_) is 301.84 min, and the maximum pump rate (*Q*_*max*_) is 10.75 μL/min. These estimates and the trend of the fitting curve closely match the experimental result. When the sucrose concentration is reduced to 3 wt%, the parameters adjust to 0.007545, 0.026433, 140.7833, and 1,002.617, respectively. Under this condition, the expected runtime is 1002 minutes, *t*_*max*_ is 263.30 min, and *Q*_*max*_ is 10.30 μL/min, and again, aligning well with the experimental data (Fig. 5). Overall, these modeling results demonstrate reliable performance for yeast and sucrose configurations.

### Application of the yeast pump

**Fig. 6:**
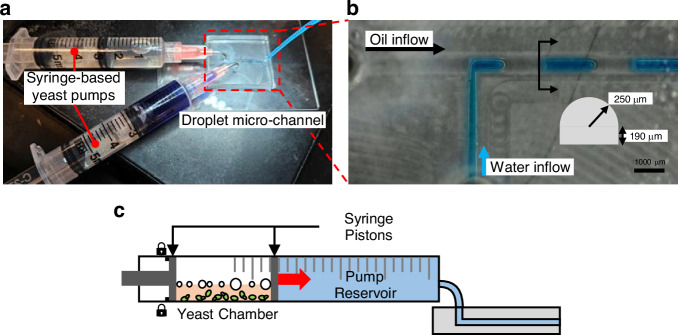
Experimental setup of an oil-water droplet generation in a microchannel operated by two yeast pumps. **a** Experiment setup using the syringe-type yeast pumps. **b** Microscope image of droplet formation (scale bar = 1000 µm). **c** Schematic of the syringe-type yeast pump

The yeast pump successfully demonstrated its capability to generate sufficient pressure to drive fluid through a microchannel (Fig. 6a, b). To verify its performance, an oil-water droplet formation experiment was conducted using a T-junction microchannel (see also Supplementary Movie S1 and S2). This channel had a semicircular cross-section with a 250 µm radius. Each pump was fabricated by modifying a commercial 10 mL syringe (Fig. 6c, Supplementary Fig. S6). Kymography was used to measure the average flow rates of 18.98 μL/min for water and 27.68 μL/min for soybean oil. Kymography is a technique that records and visualizes motion over time, typically used to study biological or mechanical data. Example images generated with kymography are shown in Supplementary Fig. S7. According to the Hagen–Poiseuille equation (Eq. (14)), approximately 29.3 Pa of pressure is required to sustain the measured flow rates in this microchannel. The variables for the equation are: pressure required to sustain the flow rate (Δ*p*), viscosity of soybean oil (*μ*), flow rate (*Q*), length of the microchannel (*L*), and radius of microchannel’s cross-section (*R*). The oil-phase pump containing 20 mg of yeast produced adequate pressure to generate droplets.

14 $$\Delta p=\frac{8\mu {QL}}{\pi {R}^{4}}$$

It is important to note that the yeast pump can generate pressures exceeding 100 kPa, far greater than the hydraulic resistance of the tested microchannel. As a result, the effect of channel geometry or fluid viscosity is negligible relative to the pump’s output capacity.

### Pump designs for utilizing yeast power

This study utilized a commercially available 10 mL syringe, which is easily accessible in most laboratories. This syringe-based system proved suitable for microfluidic experiments, and this model was fitted accordingly. However, the curve fitting parameters depend on factors such as yeast strain, pump geometry, and materials properties, because yeast fermentation rate, piston friction, and chamber volume all influence internal pressure and flow rate. Once the model is fitted, these factors can be treated as constant, but calibration is required for each yeast strain and pump design. Based on the results presented here, the parameters exhibit a linear correlation with pump configurations (yeast and sucrose), suggesting that adapting the model to new setups requires at least three calibrations to refit the curve-fitting parameters. This foundation highlights the flexibility of yeast-driven pumping, opening the way for diverse design variations that build on the same principles.

**Fig. 7:**
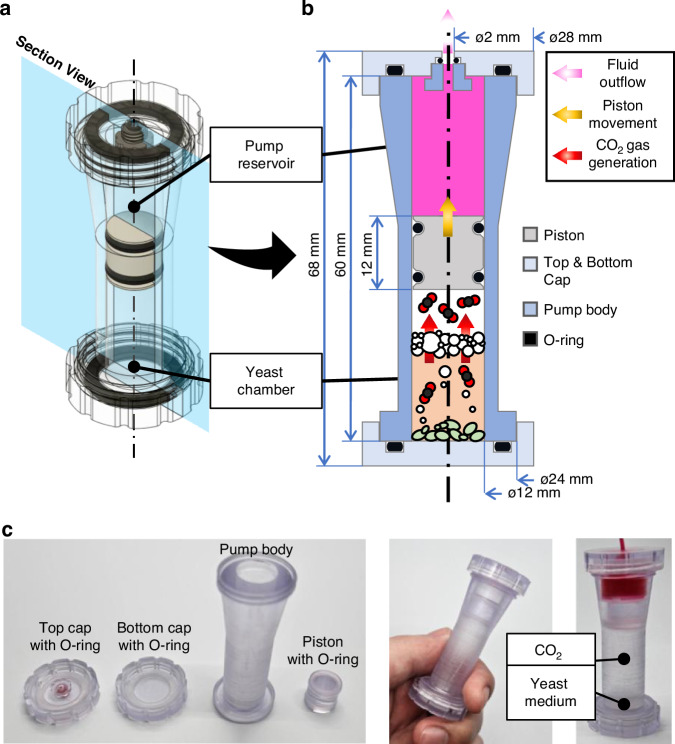
Schematic illustration and fabricated parts of yeast pump. **a** 3D modeling of the yeast pump, with a translucent blue plane indicating the sectional view. **b** Cross-section schematic and detailed dimensions of the pump. All components, except for the silicon O-ring, are fabricated from transparent polymer material using a DLP 3D printer. **c** Photograph of the fabricated components, arranged from left to right: outlet cap with O-ring, bottom cap, pump body, and piston with O-ring. Fully assembled and operating versions of the pump are also shown

The yeast pump concept is compatible with 3D printing methods, enabling tailored designs with specific media volumes and flow rates. The core design concept of the yeast pump involves separating the yeast chamber from the pump reservoir. The yeast chamber must be sealed airtight to prevent CO_2_ leakage into the reservoir. A cylindrical configuration could be adopted to support an intuitive operating mechanism, wherein CO_2_ generated by fermentation in the yeast chamber drives a piston that, in turn, displaces the solution in the pump reservoir upward (Fig. 7a). The yeast pump is composed of four main parts (Fig. 7b). An O-ring is placed between each section to ensure sealing, and all components—except the O-ring—were fabricated using Biomed Clear resin (Formlabs Inc., Somerville, MA, USA). This resin is autoclavable for sterilization, making it well-suited for use in yeast-based systems. A piston is housed within the pump body, with the lower portion forming the yeast chamber and the upper portion serving as the pump reservoir. Both compartments are sealed with caps containing O-rings to maintain airtightness. Notably, the upper section of the pump body was designed to be thicker to visually and structurally distinguish it from the lower section. Additionally, the top cap includes an insertion port equipped with an O-ring, allowing easy attachment and removal of tubing with an outer diameter of 2.5 mm, thereby maximizing usability (Fig. 7c). The top and bottom caps, along with the pump body, are threaded to allow tool-free assembly by hand. The activation of the pump is demonstrated in Supplementary Movie S3.

**Fig. 8:**
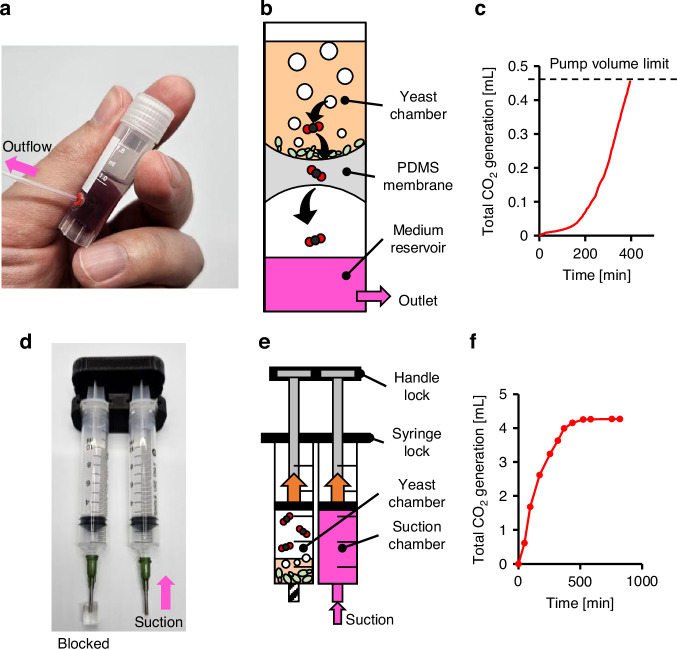
Alternative utilization of yeast-based fermentation for pumping. **a** Photograph of a diffusion-based yeast pump. **b** A gas-permeable material such as PDMS enables selective CO_2_ diffusion from the sealed yeast chamber, generating external pressure. **c** Owing to the slow diffusion rate and compact reservoir volume, this pump demonstrates limited flow rate and capacity. **d** Photograph of a syringe-based suction yeast pump. **e** In this configuration, CO_2_ expansion drives the piston in reverse, drawing fluid into a connected microfluidic system. **f** This suction-based mechanism is functionally similar to the yeast pump presented in this paper and demonstrates comparable flow rate behavior over time

Another approach uses gas-permeable membranes—such as PDMS—to implement a diffusion-based pump (Fig. 8a). In this configuration, CO_2_ produced in a sealed yeast chamber diffuses through the membrane and exerts pressure on a separate reservoir, thereby displacing fluid through the outlet (Fig. 8b). This design eliminates the need for moving mechanical components, making it particularly suitable for applications requiring simplicity, reliability, or compact integration. However, because CO_2_ diffuses slowly through PDMS, the pumping rate is significantly lower than that of the piston-driven system under comparable conditions (Fig. 8c).

The last configuration inverts the pressure direction to create a suction-based pump, where expanding gas displaces a piston to generate negative pressure, thereby drawing fluid into a connected microfluidic device (Fig. 8d, Supplementary Movie S4). Although functionally similar to the primary pump described in this study, this inverted system operates in reverse (Fig. 8e, f). It may be advantageous in integrated systems where direct fluid displacement is less desirable, or when fluid must be drawn from closed channels—such as setups with multiple inlets controlled via negative pressure at a single outlet.

Together, these configurations highlight the potential of yeast-powered pumping systems. The principle demonstrated in this study—using biologically generated gas for fluid actuation—can be adapted to various formats beyond the piston-based design introduced here.

## Conclusion

Despite its long history of use in human civilization, yeast has rarely been utilized with such precision and engineering intent for microfluidic pumping applications. The yeast-powered microfluidic pump introduced in this study represents a promising innovation in fluid manipulation technology, offering a biologically driven, autonomous, and energy-efficient alternative to conventional pumping systems. To achieve stable and predictable performance, the pump was designed using a simplified medium—only sucrose and water—to suppress active yeast reproduction and reduce variability.

We identified three characteristic operational phases—startup, stable, and decline—and developed a simplified mathematical model to describe the overall behavior of the pump. Drawing on fundamental biological and physical insights into yeast metabolism and gas generation, each phase was modeled with sigmoidal or exponential functions. The resulting composite equation, defined by four parameters, accurately captured experimental trends within the validated operating range (yeast inoculum mass ≤ 5 mg, sucrose concentration ≤ 5%). Application of the model beyond these conditions requires additional calibration and validation. Through parameter analysis under various experimental conditions, we demonstrated how each parameter influences the peak time, pump rate, and runtime. The four-parameter model can be simplified to a two-parameter form—yeast mass and sucrose concentration—through interpolation. This simplified model accurately predicts performance for yeast masses below 5 mg and sucrose concentrations below 5 wt%.

We recognized that unintended leakage of yeast could cause undesirable consequences, both mechanically and biologically. To resolve these concerns, the yeast chamber was designed to remain fully sealed throughout operation, keeping yeast and CO_2_ isolated from the external environment. With this containment strategy in place, we also developed alternative pump types, such as diffusion-driven and suction-based systems. Based on the findings presented here, the system may also be scaled up for macrofluidic applications, extending the utility of yeast-powered pumping beyond microfluidics. Moreover, the minimalistic design and biological foundation of the yeast-powered microfluidic pump provide key advantages in weight, energy efficiency, and operational reliability. These attributes make it a strong candidate for use in extreme or resource-limited environments, including space missions, remote field operations, and emergency power backups. Its self-regulating mechanism reduces the need for external control or maintenance, enhancing reliability under variable and demanding conditions. This opens promising pathways for compact, energy-efficient systems capable of autonomous fluidic operation in extraterrestrial habitats, long-duration spaceflights, battlefield deployments, and other harsh settings. Future work may focus on improving the runtime of the pump and integrating it into multifunctional lab-on-a-chip platforms for in situ biological and chemical analyses in extreme conditions, as well as aligning with advanced military technologies requiring robust, lightweight fluid handling.

## Materials and methods

### Pump fabrication and materials

The syringe-based yeast pump was fabricated using two syringes. The plunger from one syringe was removed, and its rubber head was cut off. This cut rubber head was inserted into another syringe, acting as a piston. The pumping medium was drawn through the tip, and the yeast suspension was injected into the yeast chamber from the back side of the syringe. Finally, the plunger was pushed in to seal the yeast chamber. More information is available in Supplementary Fig. S5.

The 3D design of the yeast pump was created using Fusion 360 (Autodesk Inc., San Rafael, CA, USA). The pump housing was fabricated with a biocompatible clear resin via resin-based stereolithography (SLA) 3D printing using the Formlabs Form 4 system. This approach enabled high fabrication precision and chemical resistance suitable for fermentation environments.

### Yeast suspension preparation

Instant dry yeast (*Saccharomyces cerevisiae*) was sourced from Bumafood Inc., and extra-pure sucrose was purchased from Samchun Chemical. Unless otherwise stated, the fermentation medium was prepared using deionized water containing 5 wt% sucrose and 5 mg of yeast. Inoculum mass and medium volume were adjusted based on experimental needs. To ensure accurate concentration, the sucrose solution was batch-prepared in 50 mL volumes. The designated amount of dry yeast was weighed and added to the sucrose solution in an empty conical tube to initiate activation. After allowing the suspension to rest at room temperature for up to 10 min, a 1 mL aliquot of the yeast-sucrose solution was transferred into the yeast chamber using a pipette.

### Temperature conditions

All experiments were conducted in a temperature-controlled incubator maintained at 37 °C to ensure consistent yeast fermentation activity.

### Kymography methods

Time-lapse images were captured every 3 minutes using a webcam installed inside the incubator. The images were processed using the Kymograph tool in ImageJ by drawing a line aligned along the pump cylinder. Kymograph data was exported to Excel with a custom macro, which reads the pixel data, where volume and velocity were calculated based on pixel displacement and time intervals. Examples of kymography results are shown in Supplementary Fig. S7.

### Curve fitting methods

The curve fitting was performed using MATLAB R2022b with the Curve Fitting Toolbox and a custom-defined function. For the 4-parameter model, the fitting function used was:

‘sf*(exp(st*(x-ti))/(1+exp(st*(x-ti))))*max(0,exp(1)-exp(x/te))’

Initial parameter guesses were set as follows: *sf* = 0.005, *st* = 0.005, *ti* = 100, and *te* = 1000 with a lower bound of 0 for all parameters to ensure convergence. The fitting employed a linear least squares method with a maximum of 400 iterations.

### Analysis methods

Pump performance was monitored using a digital time-lapse imaging system. A digital camera captured piston displacement at fixed intervals, and the resulting image sequences were processed using ImageJ/Fiji software (NIH, USA) to generate kymographs. Displacement data were extracted from these kymographs to quantify the temporal flow rate and cumulative displacement^31^.

Yeast cell viability was evaluated using methylene blue staining. After the base experiment, a 0.1 wt% methylene blue solution was mixed with an equal volume of the yeast-containing medium in a 1:1 ratio. The mixture was incubated at room temperature for 10 minutes. The samples were then examined using a hemocytometer and an optical microscope. Stained (nonviable) and unstained (viable) cells were counted to calculate overall cell viability.

## Supplementary information

Supplementary information

Movie S1

Movie S2

Movie S3

Movie S4

## Data Availability

All data are available in the main text or the supplementary materials.
