# Supplementary material for: Yeast-powered microfluidic pump based on a four-parameter fermentation model
Source: Microsyst Nanoeng. 2026 May 15;12:182. doi: 10.1038/s41378-026-01294-1 (PMC13179319; doi:10.1038/s41378-026-01294-1)
Supplement: Supplementary file 1 — Supplementary information [file 41378_2026_1294_MOESM1_ESM.docx]

Supporting Information

**Yeast-powered microfluidic pump based on a four-parameter fermentation model**

*Jeongmok Kim^1^, Kideok Kim^1^, Seongyeol Baeck^1^ and Joong Yull Park^1,2^**

^1^ School of Mechanical Engineering, College of Engineering, Chung-Ang University, 84 Heukseok-ro, Dongjak-gu, Seoul 06974, Republic of Korea

^2^ Department of Intelligent Energy and Industry, Graduate School, Chung-Ang University, 84 Heukseok-ro, Dongjak-gu, Seoul 06974, Republic of Korea

Correspondence*: Joong Yull Park ([jrpark@cau.ac.kr](mailto:jrpark@cau.ac.kr))

| Duplicates | | Volume (mL) | | | | | |
| --- | --- | --- | --- | --- | --- | --- | --- |
|  |  | 1 | 2 | 3 | 4 | 5 | 6 |
| Inoculum mass (mg) | 1 | 2.85 | 3.07 | N/A | 2.26 | 2.86 | 2.71 |
|  | 2 | 5.42 | 5.95 | 5.44 | 4.7 | N/A | 5.79 |
|  | 3 | 8.86 | 8.89 | 8.54 | 8.64 | 8.06 | 8.06 |
|  | 4 | N/A | 8.43 | 9.3 | 9.12 | 9.23 | 8.36 |
|  | 5 | 9.87 | 9.54 | N/A | 9.66 | N/A | 10.09 |
|  | 6 | 10.44 | 10.26 | 10.82 | 9.95 | 10.26 | 10.32 |
|  | 7 | 10.03 | 10.66 | 10.43 | N/A | 9.99 | N/A |
| Sucrose concentration (%wt) | 1 | 1.46 | 1.54 | 1.48 | 1.46 | 1.5 | 1.46 |
|  | 2 | 2.95 | N/A | 2.92 | 2.95 | 2.73 | 2.75 |
|  | 3 | 5.53 | 5.93 | 6.03 | 5.8 | 5.28 | 5.83 |
|  | 4 | 7.66 | 6.6 | 7.23 | 7.4 | 6.61 | 7.46 |
|  | 5 | 10.18 | 9.33 | 10.03 | 10.23 | N/A | 10.24 |
|  | 10 | 11 | 11.06 | 10.58 | 11.11 | 11.22 | 11.43 |

**Table S1. Total volume generated in case study experiments.**


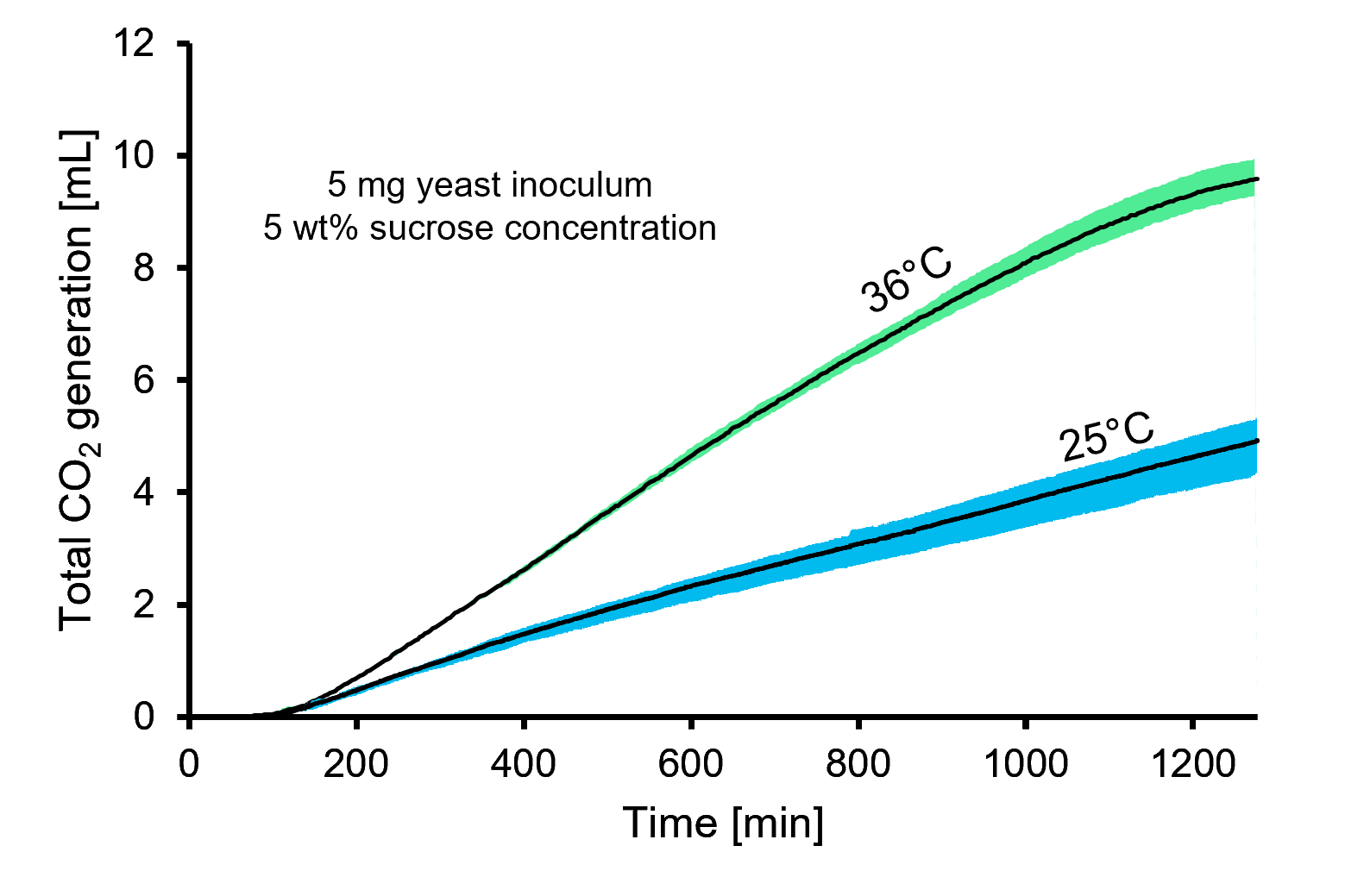


Figure S1. Cumulative CO_2_ production over time at 25°C and 36°C. The yeast pump at 25°C was found to be approximately twofold slower than values reported at 36°C, while the overall performance curve trend was preserved.


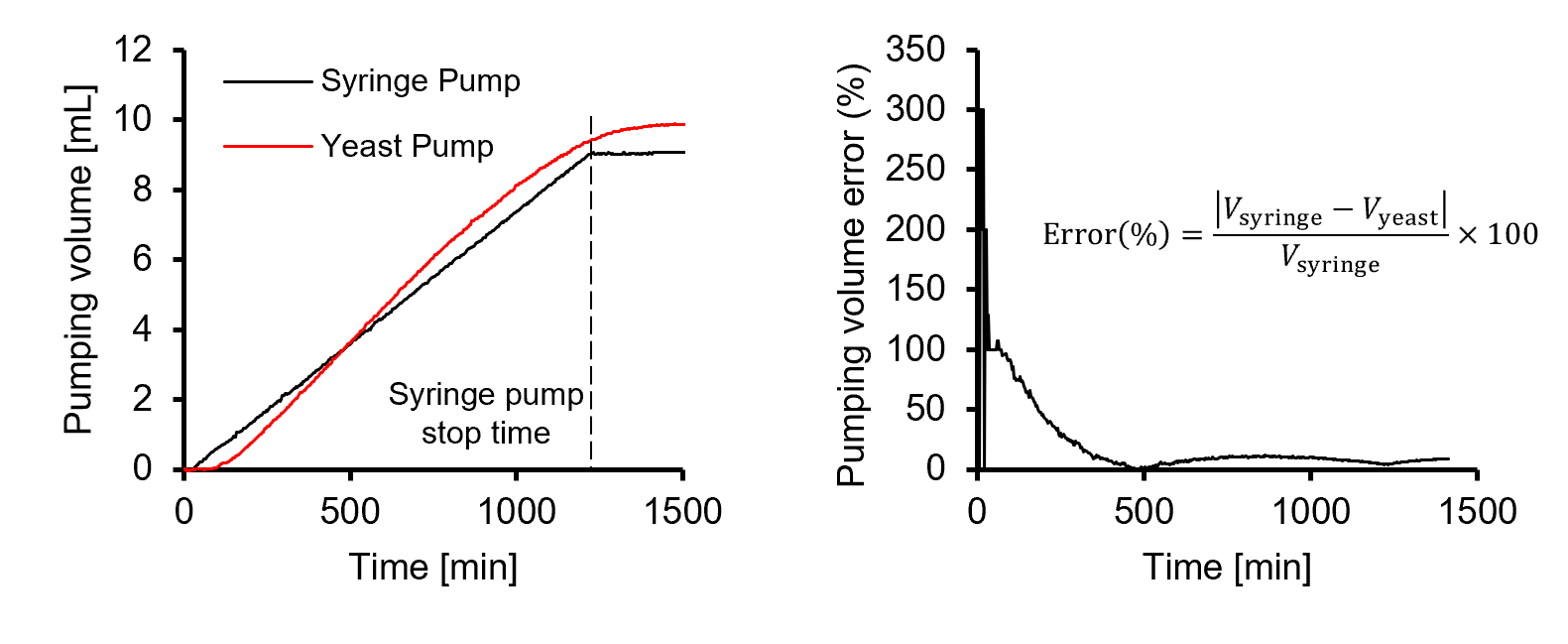


Figure S2. The yeast pump exhibited a more dynamic pumping rate than the steady linear flow rate of the syringe pump. When using the syringe pump as a reference for ideal linear flow, the yeast pump showed a relative error over 300% at the beginning, which decreased to less than 10% after 360 minutes. Due to this unique dynamic behavior, constructing a model to guide its calculation and setup is essential for general application.


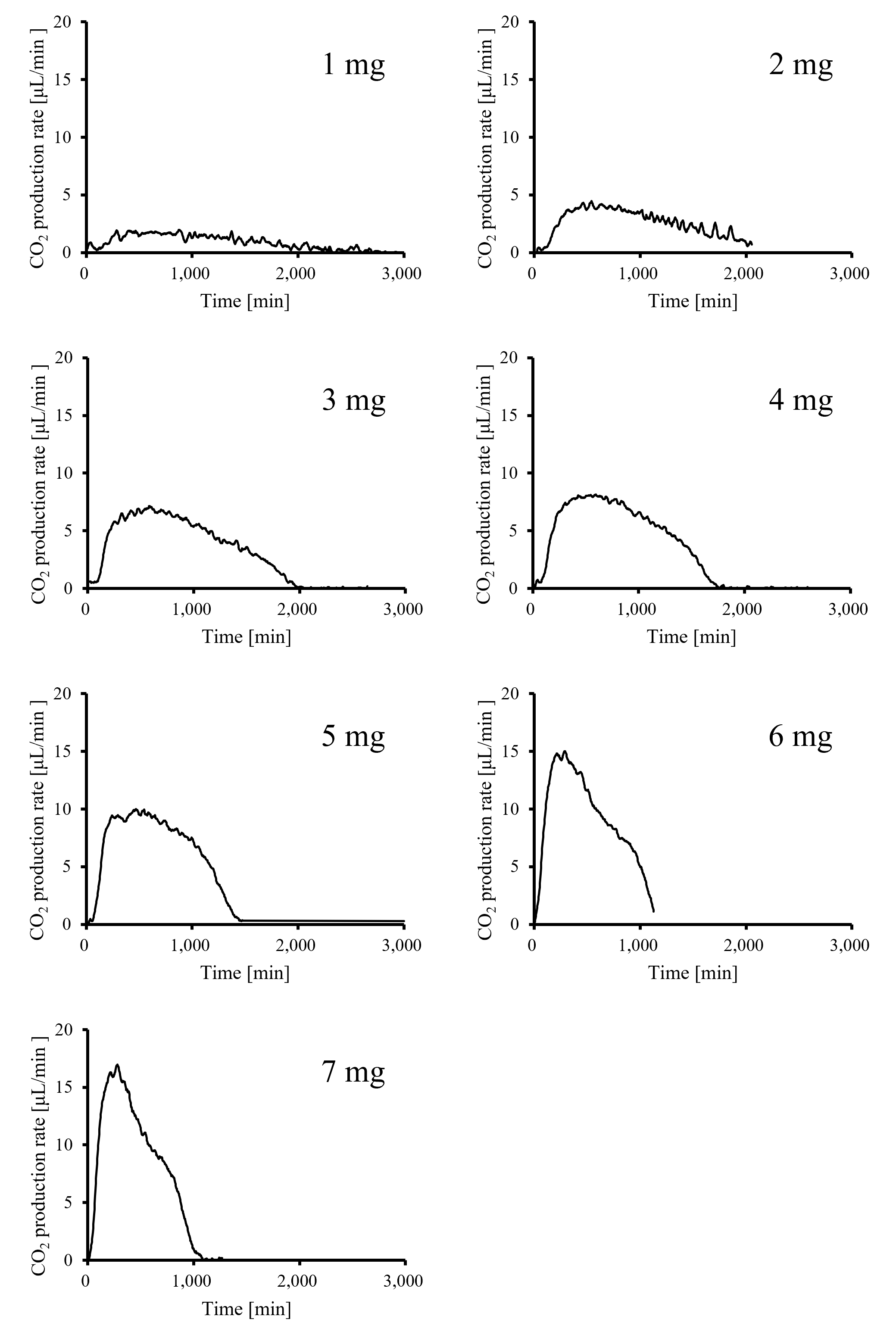


Figure S3. CO_2_ production rate curves for all inoculum mass experiments.


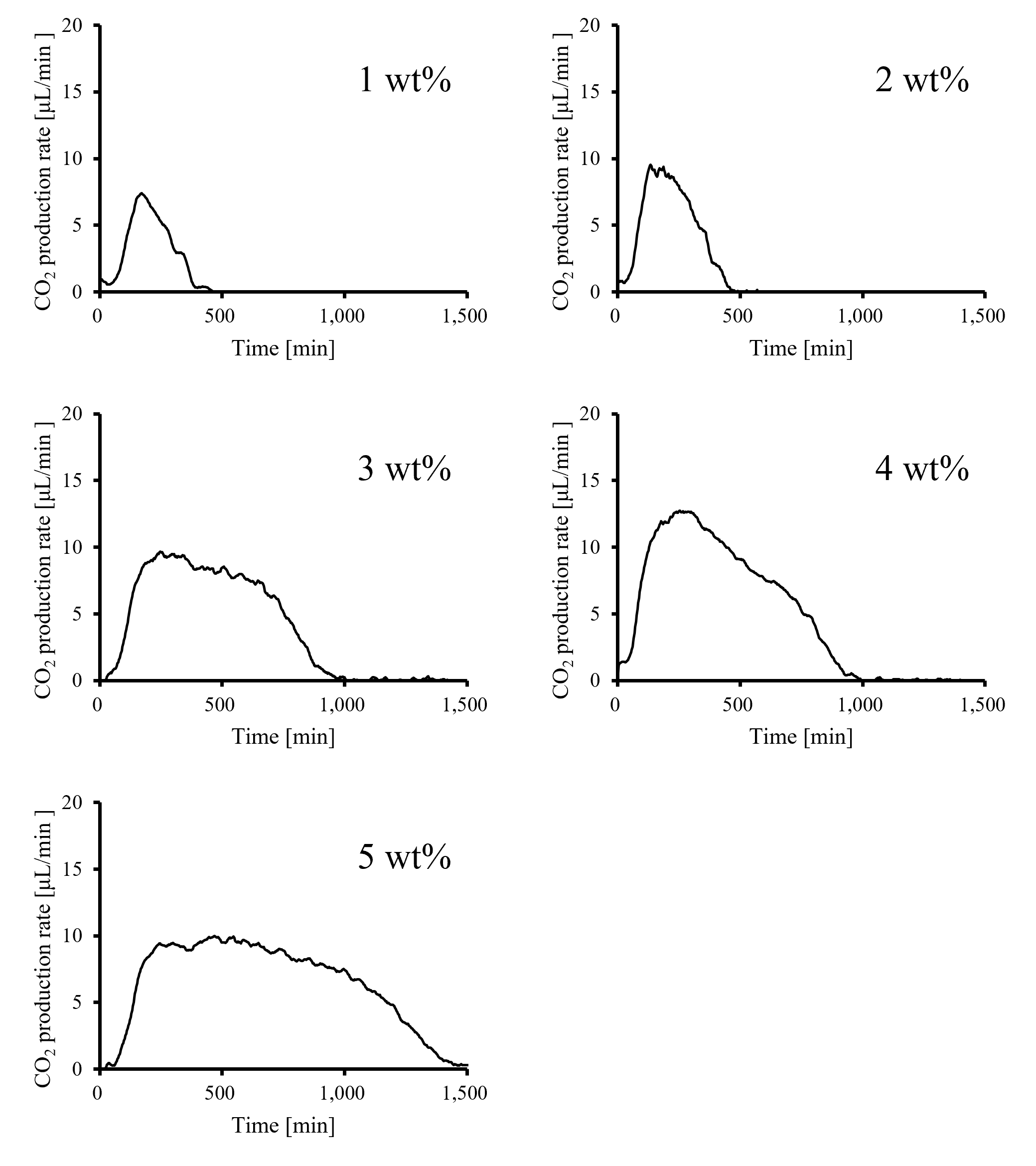


Figure S4. CO_2_ production rate curves for all sucrose concentration experiments.


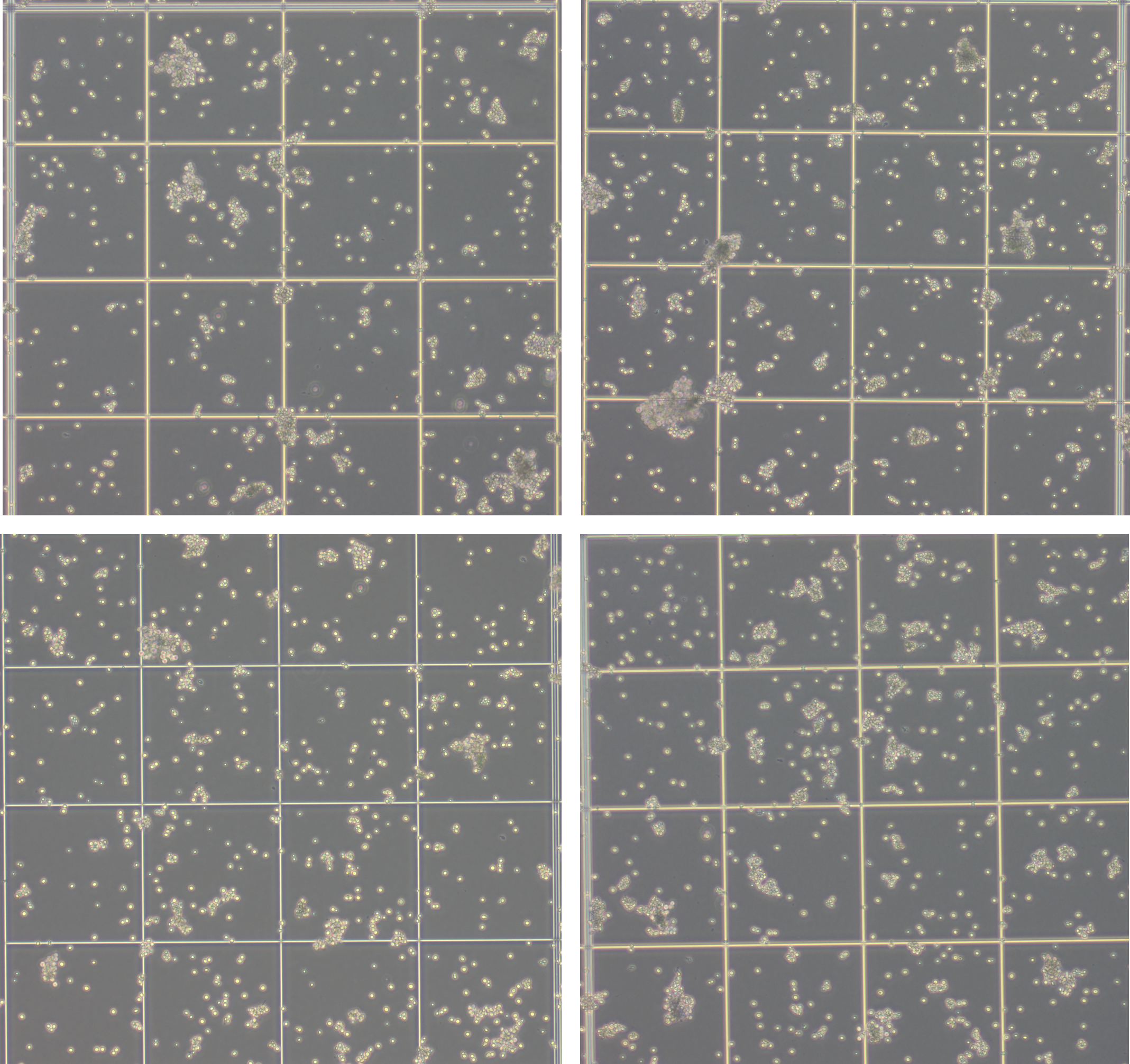

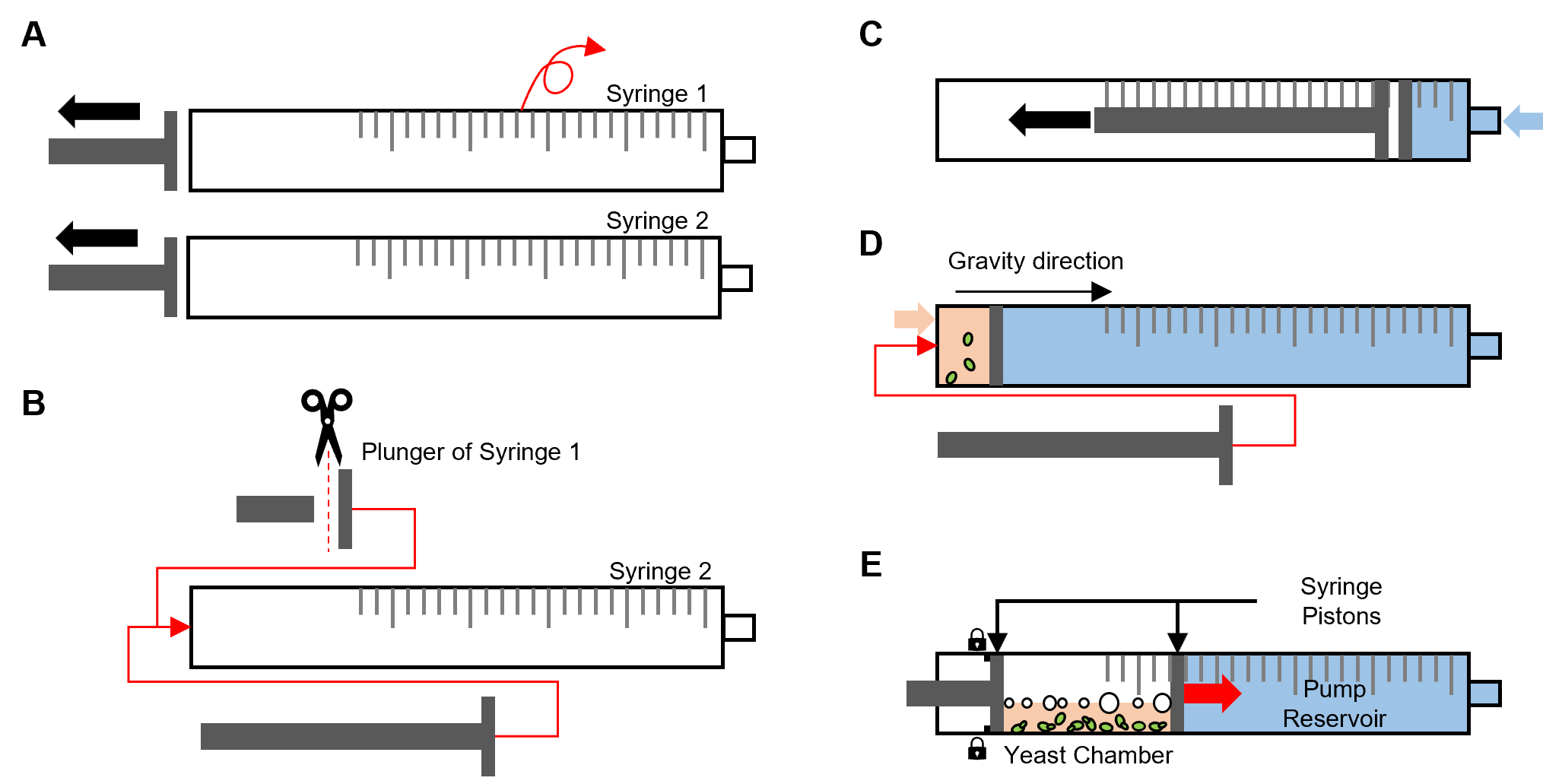


Figure S5. Viability test for base case (5 mg yeast inoculum, 5 wt% sucrose). Yeast cells were stained with 0.1 wt% methylene blue at a 1:1 volume ratio immediately after the pump experiments. Samples from six different experiments were randomly distributed across four cytometry slides, each containing eight counting grids, resulting in a total of 32 microscope images. Cell viability was assessed by counting dark-stained dead cells, and over 99% of the yeast cells remained viable following the pump experiments. The distance between the grid line in the image is 250 μm.

Figure S6. Assembly steps for the syringe-based pump. The syringe-based pump needs two identical syringes, and 10 mL was used in this paper. (A) Fully remove both plungers from the syringes. (B) Cut off the rubber head from one plunger to create a piston. Insert this piston into the second syringe and push it toward the tip with the plunger. (C) Draw the pumping fluid through the tip until the plunger is ejected from the syringe. (D) Push the piston inward to make a room for the yeast chamber; 1 mL was used in this study. (E) Reinsert the plunger and anchor its position to seal the chamber.


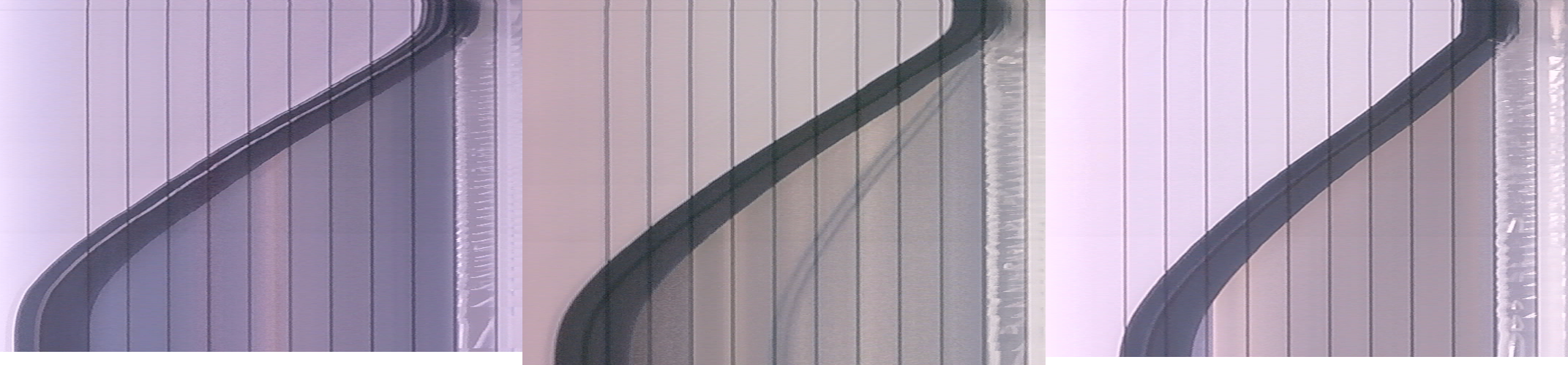


Figure S7. Example images from the case study using a 5 mg yeast inoculum and 5 wt% sucrose (N=3). The camera captured frames at intervals of 3 minutes. Data were extracted from the same position (a line in this case) in each frame and merged vertically to construct the kymograph. In this representation, horizontal pixels correspond to piston movement (discharged volume), while the vertical axis represents time. The black curve traces piston movement inside the syringe, and vertical black lines mark 1 mL intervals, which were used to calibrate and measure distance.

**Equation S1. Calculating t_max_**

$$Q_{\mathrm{total}}\left( t \right)$$

$$=s_{f}\left( \frac{e^{s_{t}\left( t-t_{i} \right)}}{1+e^{s_{t}\left( t-t_{i} \right)}} \right)\left( e-\exp\left( \frac{t}{t_{e}} \right) \right)\left\{ 0\leq t\leq t_{e} \right\}$$

$$\frac{dQ_{\mathrm{total}}}{dt}$$

$$=-s_{f}\frac{\exp\left( s_{t}\left( t-t_{i} \right) \right)\left( \exp\left( t\left( s_{t}+t_{e}^{-1} \right)-s_{t}t_{i} \right)+s_{t}t_{e}\left( \exp\left( t\cdot t_{e}^{-1} \right)-e \right)+\exp\left( t\cdot t_{e} \right) \right)}{t_{e}\left( \exp\left( s_{t}\left( t-t_{i} \right) \right)+1 \right)^{2}}$$

To find the maximum point, derivative should be 0.

$$\exp\left( t\left( s_{t}+t_{e}^{-1} \right)-s_{t}t_{i} \right)$$

$$+s_{t}t_{e}\left( \exp\left( t\cdot t_{e}^{-1} \right)-e \right)+\exp\left( t\cdot t_{e} \right)=0$$

$$t_{max}=t_{i}+\frac{1}{s_{t}}\ln\left( \left( \exp\left( 1-\frac{t_{max}}{t_{e}} \right)-1 \right)s_{t}t_{e}-1 \right)\approx t_{i}+\frac{1}{s_{t}}\left( s_{t}t_{e}-1 \right)$$

This approximation is possible because the value of term $\left( \exp\left( 1-\frac{t}{t_{e}} \right)-1 \right)$ is approximately 1, assuming that the peak time t_max_ typically occurs around 20–40% of t_e_.
